# C/EBPβ and YY1 bind and interact with Smad3 to modulate lipopolysaccharide‐induced amelotin gene transcription in mouse gingival epithelial cells

**DOI:** 10.1002/2211-5463.12566

**Published:** 2018-12-27

**Authors:** Yohei Nakayama, Ryoki Kobayashi, Yasunobu Iwai, Keisuke Noda, Mizuho Yamazaki, Tomoko Kurita‐Ochiai, Atsutoshi Yoshimura, Bernhard Ganss, Yorimasa Ogata

**Affiliations:** ^1^ Department of Periodontology Nihon University School of Dentistry at Matsudo Chiba Japan; ^2^ Research Institute of Oral Science Nihon University School of Dentistry at Matsudo Chiba Japan; ^3^ Department of Oral Immunology Nihon University School of Dentistry at Matsudo Chiba Japan; ^4^ Department of Periodontology Nagasaki University Graduate School of Biomedical Sciences Japan; ^5^ Matrix Dynamics Group Faculty of Dentistry University of Toronto Canada

**Keywords:** amelotin, gene promoter, inflammation, junctional epithelium, lipopolysaccharide, periodontitis

## Abstract

Junctional epithelium (JE) develops from reduced enamel epithelium during tooth formation and is critical for the maintenance of healthy periodontal tissue through ensuring appropriate immune responses and the rapid turnover of gingival epithelial cells. We have previously shown a relationship between inflammatory cytokines and expression of JE‐specific genes, such as amelotin (AMTN), in gingival epithelial cells. Here, we elucidated the effects of *Porphyromonas gingivalis*‐derived lipopolysaccharide (*Pg* LPS) on *Amtn* gene transcription and the interaction of transcription factors. To determine the molecular basis of transcriptional regulation of the *Amtn* gene by *Pg* LPS, we performed real‐time PCR and carried out luciferase assays using a mouse *Amtn* gene promoter linked to a luciferase reporter gene in mouse gingival epithelial GE1 cells. Gel mobility shift and chromatin immunoprecipitation assays were performed to identify response elements bound to LPS‐induced transcription factors. Next, we analyzed protein levels of the LPS‐induced transcription factors and the interaction of transcription factors by western blotting and immunoprecipitation. LPS increased *Amtn* mRNA levels and elevated luciferase activities of constructs containing regions between −116 and −238 of the mouse *Amtn* gene promoter. *CCAAT*/enhancer‐binding protein (*C/EBP*) 1–, *C/EBP2*– and Ying Yang 1 (*YY1*)–nuclear protein complexes were increased by LPS treatment. Furthermore, we identified LPS‐modulated interactions with C/EBPβ, YY1 and Smad3. These results demonstrate that *Pg* LPS regulates *Amtn* gene transcription via binding of C/EBPβ–Smad3 and YY1–Smad3 complexes to C/EBP1, C/EBP2 and YY1 response elements in the mouse *Amtn* gene promoter.

**Abbreviations**
ALK5: activin receptor‐like kinase 5
AMTN: amelotin
C/EBP: CCAAT/enhancer‐binding protein
ChIP: chromatin immunoprecipitation
EGF: epidermal growth factor
FDC‐SP: follicular dendritic cell‐secreted protein
GAPDH: glyceraldehyde 3‐phosphate dehydrogenase
HDAC1: histone deacetylase‐1
IL‐1β: interleukin‐1β
JE: junctional epithelium
LPS: lipopolysaccharide
ODAM: odontogenic ameloblast‐associated protein
*Pg*: *Porphyromonas gingivalis*
PI3‐K: phosphatidylinositol 3‐kinase
PKA: protein kinase A
RT: room temperature
SBE: Smad binding elements
TGF‐β1: transforming growth factor β1
TNF‐α: tumor necrosis factor‐α
V1: variant 1
V2: variant 2
YY1: Ying Yang 1

Junctional epithelium (JE) is developed from reduced enamel epithelium during tooth formation 1, 2 and persists on the tooth surface, including on mature enamel and cementum with gingival recession. Internal basal laminae of JE and tooth surfaces are bound to each other by hemidesmosomes. The seal of periodontal tissue prevents the entry of bacteria and products such as lipopolysaccharide (LPS), subsequently produced proinflammatory cytokines and proteolytic enzymes 3. Consequently, it is widely accepted that the function of JE is critical for the maintenance of healthy periodontal tissue by an appropriate immune response and the rapid turnover of gingival epithelial cells 4.

We have previously shown a relation between inflammatory cytokines and expression of JE‐specific genes, such as amelotin (AMTN) 5, 6, 7, 8, odontogenic ameloblast‐associated protein (ODAM) and follicular dendritic cell‐secreted protein (FDC‐SP) 9, 10, 11, during dental development and regeneration of JE in gingival epithelial cells and a *Porphyromonas gingivalis* (*Pg*)‐ and *Aggregatibacter actinomycetemcomitans*‐infected mouse periodontitis model 12. Moreover, we have reported that *Amtn* gene expression was temporarily increased via Smad3 binding to Smad binding elements (SBEs) at the initiation of transforming growth factor β1 (TGF‐β1)‐induced apoptosis in gingival epithelial cells 13, 14.

*Amtn* genes from mouse and human display high homologous exon–intron structure and were expressed from loci on chromosomes 5 and 4, respectively, which have a close proximity to enamelin (*ENAM*), ameloblastin (*AMBN*) and the small, integrin‐binding ligand N‐linked glycoprotein (SIBLING) gene family 15. The AMTN protein is enriched in glutamine, leucine, proline and threonine (52% of total) and contains a conserved protein kinase (casein kinase 2) phosphorylation site 16. *Amtn* transcripts demonstrated to have two variants, variant 1 (V1) and variant 2 (V2). V1 represented 70.8% of *Amtn* transcripts and displayed the structure known in rodents, whereas V2 represented 29.2% and exhibited the non‐mammalian tetrapod structure 17. Differences of the function of AMTN protein due to *Amtn* gene structure are unclear, though AMTN protein has a direct influence on biomineralization by promoting hydoroxyapatite (HA) mineralization 18 and is associated with amelogenesis imperfecta 19.

AMTN and ODAM are expressed not only by mature ameloblasts at amelogenesis but also in JE at an erupted tooth 16, 20, 21. AMTN knockout mice showed hypomature enamel 22, and AMTN overexpression mice have irregular enamel structures and thin enamel compared with wild‐type mice 23; however, both mice exhibited no pronounced abnormality of periodontium. Whereas ODAM‐deficient mice showed gingival recession with aging, delay in regeneration following gingivectomy and a tendency towards decrease of AMTN expression in JE, no irregular enamel structures were observed 24. It has been stated that AMTN, ODAM and FDC‐SP form complexes at JE of erupted tooth surfaces 25, 26. AMTN and FDC‐SP localize at internal basal lamina of JE, whereas ODAM is evenly distributed in JE 27. Therefore, it is important to understand the mechanism of proper maintenance of JE structure by investigation the regulatory mechanism of *Amtn* gene transcription in inflammation.

*Porphyromonas gingivalis* (*Pg*) is a major periodontopathic bacterium 28. LPS is one of the multiple virulence factors in the Gram‐negative bacterium and therefore might contribute to the initiation and progression of periodontal diseases 29, 30. In our previous studies, tumor necrosis factor‐α (TNF‐α) and interleukin‐1β (IL‐1β) stimulate human *Amtn* gene transcription via *CCAAT* enhancer‐binding protein (*C/EBP*) and Ying Yang 1 (*YY1*) elements 7, 8, 31. The relationship between C/EBPs and inflammation has been reported 32 and YY1 is also a transcription factor associated with inflammation and cancer 33. However, the regulatory mechanism of *Amtn* gene transcription by *Pg*LPS, which can induce TNF‐α and IL‐1β in inflamed gingiva, remains to be elucidated.

Here, we report that *Pg*LPS‐induced physical interactions between C/EBPβ, YY1 and Smad3 upregulate *Amtn* gene transcription via mitogen‐activated protein kinase kinase 1/2 (MEK1/2), phosphatidylinositol 3‐kinase (PI3‐K) and Smad2/3 pathways in mouse gingival epithelial cells. These findings raise the possibility of an association between induced C/EBPβ and YY1 in inflammation and a constitutive interaction with Smad3 in gingival epithelial cells.

## Materials and methods

### Antibodies and regents

SFM‐101 medium was obtained from Nissui (Tokyo, Japan). Fetal calf serum (FCS), penicillin, streptomycin, TrypLE™ Express and Lipofectamine 2000 were purchased from Invitrogen (Carlsbad, CA, USA). PGL3‐basic, pSV‐β‐galactosidase (β‐Gal) control vector and U0126 (MEK1/2 inhibitor) were purchased from Promega Co. (Madison, WI, USA). Epidermal growth factor (EGF), phenylmethylsulfonyl fluoride (PMSF) and KT5720 (a PKA inhibitor) were purchased from Sigma‐Aldrich Japan (Tokyo, Japan). LY249002 (a PI3‐K inhibitor) was purchased from Calbiochem (San Diego, CA, USA) and SB525334 [TGF‐β1 receptor (an activin receptor‐like kinase 5; ALK5) inhibitor] was purchased from Wako (Osaka, Japan). Isogen II was purchased from Nippon Gene (Tokyo, Japan). The Script RT reagent Kit and SYBR Premix Ex Taq and PrimeScript RT Reagent Kit and SYBR Premix Ex Taq II were purchased from TaKaRa (Tokyo, Japan). The QuikChange Site‐Directed Mutagenesis Kit was purchased from Agilent Technologies (Santa Clara, CA, USA). KAPA Taq™ EXtra HotStart was purchased from Kapa Biosystems (Boston, MA, USA). ECL Prime Western Blotting Detection Reagent was purchased from Bio‐Rad (Tokyo, Japan). Protein A/G PLUS‐Agarose Immunoprecipitation Regent (sc‐2003) was obtained from Santa Cruz Biotechnology (Dallas, TX, USA). *Pg*LPS was purified as described previously 34.

### Cell cultures

A mouse‐derived gingival epithelial cell line (RCB1709; GE1) was purchased from Riken BRC (Ibaraki, Japan) 35; cells were cultured at 33 °C in 5% CO_2_/95% air in SFM‐101 containing 1% FCS, 10 ng·mL^−1^ EGF and 1% penicillin and streptomycin. Cells were grown to confluence in 60 mm cell culture dishes for RNA extraction; the media were then changed to no serum and EGF SFM‐101 medium for 24 h. Then, cells were incubated in this medium with or without *Pg*LPS (0.1 μg·mL^−1^) for 0, 12 and 24 h. For the extraction of nuclear protein and DNA–protein complex, 100 mm cell culture dishes were used.

### Real‐time PCR

Total RNA was isolated using Isogen II from the GE1 cell cultures, and was used as a template for cDNA synthesis with an ExScript RT reagent kit (TaKaRa). Quantitative real‐time PCR was performed using the following primer sets: mouse *Amtn* forward, 5′‐CTGTCAACCAGGGAACCACT‐3′; mouse *Amtn* reverse, 5′‐TGTGATGCGGTTTAGCTGAG‐3′; and mouse glyceraldehyde 3‐phosphate dehydrogenase (*gapdh*) forward, 5′‐TGAAGGGGTGGTTGATGG‐3′; mouse *gapdh* reverse, 5′‐A AATGGTGAAGGTCGGTGTG‐3′. SYBR Premix Ex Taw was used in a TP800 thermal cycler dice real‐time system (TaKaRa). Primers were purchased from Sigma‐Aldrich Japan. To investigate the signaling pathways in the transcriptional regulation of the *Amtn* gene by *Pg*LPS, we used protein kinase inhibitors, such as KT5720, U0126, LY249002 and SB525334. After the GE1 cells were at confluence, the cells were deprived of FCS for 12 h, and first treated with KT5720 (100 nm), U0126 (5 μm), LY249002 (10 μm) and SB525334 (1 μm) for 30 min, and then incubated with or without *Pg*LPS (0.1 μg·mL^−1^) for 24 h before harvesting. The amplification reactions were performed in 25 μL of the final reaction mixture containing: 2 × SYBR Advantage qPCR Premix or SYBR Premix EX Taq (12.5 μL); 10 μm forward and reverse primers (final concentration was 0.2 μm); 50 ng (5 μL) cDNA for *Amtn* and 20 ng (2 μL) cDNA for *gapdh*. To reduce variability between replicates, PCR premixes that contained all reagents except for cDNA, were prepared and aliquot into 0.2 mL Hi‐8‐tubes (TaKaRa). The thermal cycling condition was 10 s at 95 °C and 40 cycles of 5 s, 95 °C and 30 s, 60 °C. Post‐PCR melting curves confirmed the specificity of single‐target amplification. The initial amount of RNA was quantified using a standard curve, and fold expressions of *Amtn* relative to *gapdh* were determined in triplicate. To visualize and confirm control levels of *Amtn* mRNA levels and appropriate amplification, running gel was performed using 8 μL PCR products. The product size is 208 bp according to design of primers.

### Transient transfection assays

GE1 cells were used for transfection assays. Forty‐eight hours after plating, cells at 50–70% confluence were transfected using a Lipofectamine 2000 reagent. The transfection mixture included 2 μg of the respective luciferase (LUC) construct (−116*AMTN*, −116 ~ +65 mouse *AMTN* gene promoter; −238*AMTN*, −238 ~ +65; −460*AMTN*, −460 ~ +65; −705*AMTN*, −705 ~ +65; −800*AMTN*, −800 ~ +65), and 1 μg β‐Gal vector as an internal control. Two days post‐transfection, cells were deprived of FCS and EGF for 12 h, and *Pg*LPS (0.1 μg·mL^−1^) was added for 24 h prior to harvesting. Luciferase activities were measured according to the supplier's protocol (PicaGene, Toyo Inki, Tokyo, Japan) using a luminescence reader (AccuFLEX Lumi400; Aloka, Tokyo, Japan). The mutant luciferase constructs mutant *C/EBP1* (−238m*C/EBP1*; GTGTcGGtAAg), mutant *C/EBP2* (−238m*C/EBP2*; ACATcGGaTAgTAC) and mutant *YY1* (−238m *YY1*; TGcCTGCAgTcTT) were made using the QuikChange Site‐Directed Mutagenesis Kit within the context of the homologous −238 ~ +60*Amtn* gene promoter fragments.

### Gel mobility shift assay

Nuclear protein extracts from GE1 cells were incubated for 12 and 24 h with *Pg*LPS (0.1 μg·mL^−1^) in SFM‐101 without serum. Cy5‐labeled double‐stranded oligonucleotide encompassing the inverted *CCAAT*,* C/EBP1*,* C/EBP2* and *YY1* elements in the mouse *Amtn* gene promoter were prepared. Their complementary oligonucleotide and Cy5‐labeled oligonucleotide were purchased (Sigma‐Aldrich Japan; Table 1), and they were annealed under optimal conditions. The following steps were carried out as described in a recent study 7, 8, 31. Briefly, nuclear proteins (20 μg) and 0.1 pm Cy5‐labeled double‐stranded oligonucleotide were combined and incubated for 20 min at room temperature (RT) in the binding buffer; then, the DNA–nuclear protein complexes were separated by electrophoresis in 5% acrylamide gels. Imaging of gels was analyzed using a Typhoon Trio+ Variable Mode Imager (GE Healthcare). For competition experiments, 40‐fold molar unlabeled oligonucleotides of inverted *CCAAT*,* C/EBP1*,* C/EBP2* and *YY1* were used 7. Supershift experiments were carried out using the appropriate unconjugated normal rabbit anti‐IgG antibody (sc‐2027, Santa Cruz, Biotechnology), and rabbit polyclonal antibodies to C/EBPβ (sc‐746; Santa Cruz Biotechnology), YY1 (chromatin immunoprecipitation (ChIP) Grade; ab38422; Abcam) and Smad3 (ChIP Grade; ab28379, Abcam, Cambridge, UK). Prior to the incubation of nuclear extracts with Cy5‐labeled double‐stranded oligonucleotide, antibodies were added to each reaction mixture and incubated for 4 h before electrophoresis.

**Table 1:** Sequences of oligonucleotides for gel mobility shift assay

Response element	Including		Sequence (5′–3′)	Location in the mouse AMTN promotor
*CCAAT* (−66 ~ −62)		For	*GATCC*TTAGTCCTGATTGGTCTCTCAGG*A*	−75 ~ −53
		Rev	*G*AATCAGGACTAACCAGAGAGTCC*TCTAG*	
*C/EBP1* (−113 ~ −103)		For	*GATCC*CTAATGAAGTGTTGGGAAATGAAACC*A*	−120 ~ −95
		Rev	*G*GATTACTTCACAACCCTTTACTTTGG*TCTAG*	
*C/EBP2* (−157 ~ −144)	*SBE2* (−159 ~ −156)	For	*GATCC*TCGTAGACATTGGGTAATACAATGTC*A*	−163 ~ −138
		Rev	*G*AGCATCTGTAACCCATTATGTTACAG*TCTAG*	
*YY1* (−221 ~ −209)		For	*GATCC*TTTTCTGTCTGCACTTTTTTAAAATT*A*	−226 ~ −200
		Rev	*G*AAAAGACAGACGTGAAAAAATTTTAA*TCTAG*	

### Chromatin immunoprecipitation assay

To support the results of gel mobility shift assay, indicating that C/EBPs, YY1 and Smad3 bind specifically to their response elements in the mouse *Amtn* gene promoter, chromatin immunoprecipitation (ChIP) assays were performed using GE1 cells. Confluent GE1 cells were treated for 12 and 24 h with *Pg*LPS (0.1 μg·mL^−1^) in SFM‐101 without serum. Prior to cell harvest, cell fixation with 160 μL formaldehyde to cross‐link the DNA–protein complexes was performed. Extraction of DNA including response elements was conducted as described previously 8. Two micrograms of the same antibodies as for the gel mobility shift assay was used. The purified DNA was subjected to PCR amplification for inverted *CCAAT*,* C/EBP1*,* C/EBP2* and *YY1* within the mouse *Amtn* gene promoter using primer sets (Table 2). For the PCR procedure, KAPA Taq™ EXtra HotStart was utilized.

**Table 2:** Primer list for ChIP assay

Primer name	Including		Length	Sequence (5′–3′)	Product Size	Location in the mouse AMTN promotor
*CCAAT* (−66 ~ −62)		For	24	GAAACCAAATGATTTCACAGACAG	100	−101 ~ −2
		Rev	24	GCTGATTTGTGTTTGTAGAAGGAA		
*C/EBP1* (−113 ~ −103)	*CCAAT* (−66 ~ −62)	For	22	CAATGTCGCAGCTAATAAC	101	−144 ~ −44
	*SBE1* (−82 ~ −79)	Rev	21	GACAGACAACCTGAGAGACC		
*C/EBP2* (−157 ~ −144)	*SBE2* (−159 ~ −156)	For	20	AAAATTATATAGGCATGTCTCCC	94	−206 ~ −113
		Rev	21	TTCATTAGGAGGGTTATTAGC		
*YY1* (−221 ~ −209)		For	21	GCAAAAGAGTTGAAAGTAAAG	103	−250 ~ −148
		Rev	20	CTACGATCTGACTAGAGGGT		

### Immunoprecipitation and western blotting

For regulation of *Amtn* gene transcription by *Pg*LPS via YY1 and Smad3, immunoprecipitation was carried out to examine whether YY1 and Smad3 binding responded independently or interactively to their own elements in the mouse *Amtn* gene promoter. Firstly, to nuclear proteins (15 μg) in NP‐40 solution (0.5% NP‐40, 10 mm Tris (pH 7.5), 0.5 m NaCl, 0.2 mm PMSF in ddH_2_O) was added 0.25 μg of unconjugated normal rabbit anti‐IgG antibody (sc‐2027, Santa Cruz Biotechnology) together with 20 μL of appropriate suspended protein A/G PLUS‐Agarose Immunoprecipitation Regent, and they were then incubated at 4 °C for 30 min with gentle rotation. After centrifugation of pellet beads, to the transferred supernatant was added 10 μL of 10% BSA and 1 μg antibodies (same as ChIP assay), and they were incubated at 4 °C for 1 h with gentle rotation. To nuclear protein–antibody complexes was added 20 μL of appropriate protein A/G PLUS‐Agarose conjugate suspension, and they were mixed and incubated at 4 °C with gentle rotation overnight. The pellet was collected by centrifugation at 30 000 ***g*** for 30 s at 4 °C; then, the supernatant was carefully removed. The flow‐through lysates were used for detection of histone deacetylase‐1 (HDAC1) expression as controls. After washing the pellet with NP‐40 solution twice, pellet was resuspended in 1 mL of 10 mm Tris/HCl buffer (pH 7.5). The final washed pellet without supernatant was resuspended in 15 μL of electrophoresis sample buffer (S3401; Sigma‐Aldrich Japan), then boiled for 5 min, and used for the western blotting procedure. The nuclear proteins that were precipitated with IgG or Smad3 were separated on 12% SDS/PAGE and transferred to a Hybond 0.2‐μm poly(vinylidene difluoride) membrane. The membrane was incubated at 4 °C overnight with anti‐YY1 (1 : 500) or anti‐C/EBPβ (1 : 1000) antibodies. Anti‐rabbit and anti‐mouse IgG conjugated with horseradish peroxidase were used as the secondary antibodies. ECL Prime Western Blotting Detection Reagent was used to detect immunoreactivities; the imaging was visualized with an ImageQuant LAS4000 Mini system (GE Healthcare Japan, Tokyo, Japan).

Anti‐YY1 (1 : 500), anti‐C/EBPβ (1 : 1000), anti‐Smad3 (1 : 3000), anti‐pSmad3 (1 : 1000; anti‐rabbit monoclonal antibody, ab52903, Abcam) and anti‐HDAC1 (1 : 1000; anti‐mouse monoclonal antibody, sc‐81598, Santa Cruz Biotechnology) antibodies were used for determining whether *Pg*LPS regulated these transcriptional factors at protein levels. Densitometric data were provided using imagej software (National Institutes of Health, Bethesda, MD, USA), according to the supplier's description. Briefly, each area of a band was detected by the software and values were calculated compared with HDAC1 expression (Table 3).

**Table 3:** Densitometric data for western analysis

	Control	*Pg*LPS 12 h	*Pg*LPS 24 h
Area of band			
C/EBPβ1	19 708.262	33 628.233	14 694.2
YY1	12 419.158	23 161.685	5834.803
Smad3	20 633.362	24 004.078	27 576.777
pSmad3	6368.238	22 590.877	19 491.927
HDAC1	29 852.999	39 631.342	32 471.17
Area of bands per each HDAC1			
C/EBPβ1	0.660176956	0.848526225	0.45253066
YY1	0.416010398	0.584428481	0.1796918
Smad3	0.691165467	0.605684208	0.849269583
pSmad3	0.213319874	0.570025537	0.600284098
HDAC1	1	1	1
Fold compared with each controls			
C/EBPβ1	1	1.285301186	0.685468731
YY1	1	1.404841041	0.431940647
Smad3	1	0.876323018	1.228750022
pSmad3	1	2.672163286	2.814009241
HDAC1	1	1	1

### Immunocytochemistry

To examine protein expression of C/EBPβ and YY1 in GE1 cells, immunofluorescence was carried out. After plating 30 μL of GE1 cells from confluence in 75 cm^2^ flask on culture slides (Falcon^®^ CultureSlides, Corning^®^ BioCoat™, Hampton, NH, USA), the plates with cells were incubated at 33 °C for 12 h. After treatment with *Pg*LPS for 12 or 24 h, the cells were rinsed with PBS twice and fixed by 4% paraformaldehyde for 15 min at RT. The fixed cells were rinsed by PBS twice and permeabilized with 0.5% Triton X‐100 in PBS for 10 min at RT. After removing permeabilization solution, there followed blocking with 1% bovine serum albumin in PBS for 20 min at RT. The cells were treated with 100 μL of primary antibodies diluted with buffer (1% BSA in PBS) for 1 h at RT followed by incubation with secondary antibodies for 1 h at RT. For the primary antibodies, we used C/EBPβ (1 : 50, sc‐746; Santa Cruz Biotechnology), YY1 (ChIP Grade; ab38422; Abcam) rabbit polyclonal antibodies and CK19 (1 : 100, ab7755, Abcam) mouse monoclonal antibody. For detection signaling for fluorescence, we used goat anti‐mouse IgG H&L (Alexa Fluor^®^ 488, 1 : 200, ab150113, Abcam) and goat anti‐rabbit IgG H&L (Alexa Fluor^®^ 647, 1 : 200, ab150079, Abcam). Mounting and nuclear staining with 4′,6‐diamidino‐2‐phenylindole (DAPI) were performed (Fluoroshield mounting medium with DAPI, ab104139, Abcam).

### Statistical analysis

Triplicate samples for real‐time PCR were analyzed for each experiment and were replicated to ensure consistency of the responses to *Pg*LPS. Significant differences between the control and treatments were determined using multiple comparison calibration (the Tukey–Kramer method) for real‐time PCR and one‐way analysis of variance (one‐way anova).

## Results

### Upregulation of *Amtn* mRNA levels by *P. gingivalis* LPS in GE1 cells

**Figure 1:**
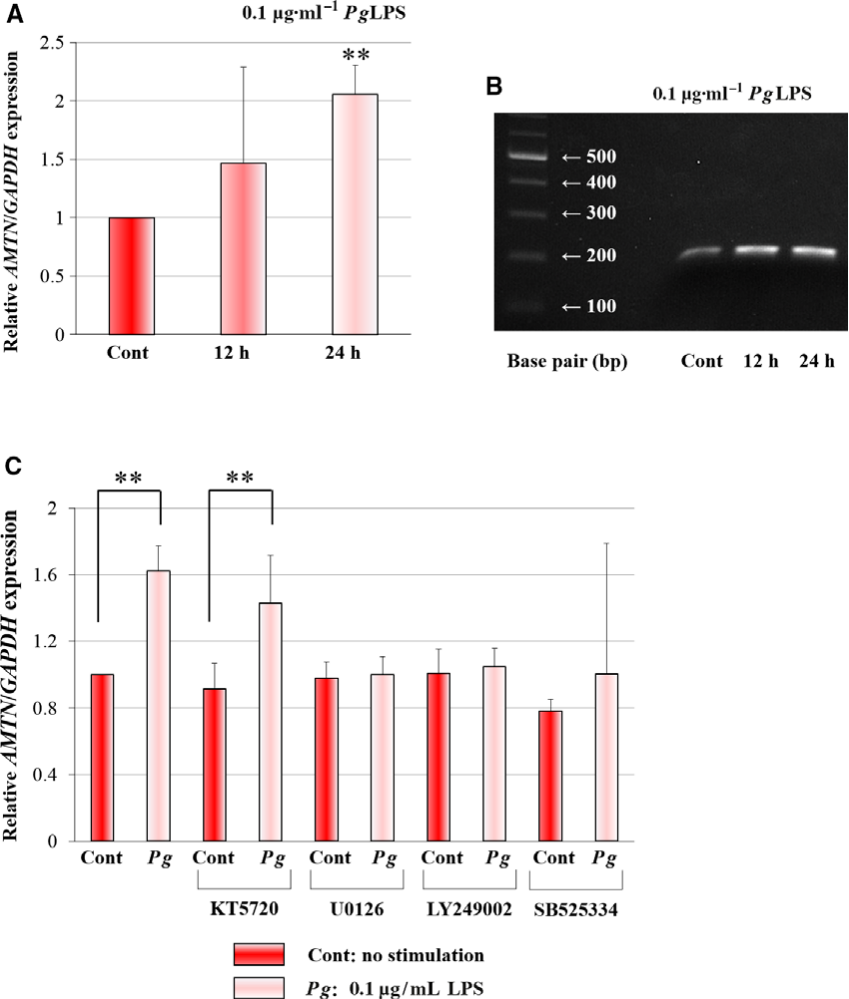
Alteration of *Amtn* mRNA levels by *Pg* LPS in mouse GE1 gingival epithelial cells. (A) Effects of *Pg* LPS (0.1 μg·mL ^−1^) on *Amtn* (A) mRNA levels at 12 and 24 h. The values are expressed with standard errors (SE). Significant differences from the control: **P <* *0.01 (*n *=* *3). (B) Evaluation of PCR products by gel loading analysis. Effects of kinase inhibitors on the induction of *Amtn* mRNA levels by *Pg* LPS. (C) Effects of PKA (KT5720), MEK1/2 kinase (U0126), PI3‐K (LY249002) and ALK5 (SB525334) inhibitors on the *Amtn* mRNA levels induced by *Pg* LPS (0.1 μg·mL ^−1^) for 24 h in GE1 cells. The values are expressed with standard errors. Significant differences from the control: **P <* *0.01 (*n *=* *3).

To determine the *Amtn* mRNA levels after treatment by *Pg*LPS (0.1 μg·mL^−1^), we performed real‐time PCR using total RNA from GE1 cells. *Amtn* mRNA levels were significantly increased by *Pg*LPS at 24 h (Fig. 1A). We confirmed increases of *Amtn* mRNA levels by appropriate amplification with visible bands in 2% agarose gel. To determine the signaling pathways for the regulation of *Amtn* gene transcription by *Pg*LPS in GE1 cells, we used protein kinase inhibitors. *Amtn* mRNA levels were significantly increased by *Pg*LPS at 24 h. U0126 (a MEK1/2 inhibitor), LY249002 (a PI3‐K inhibitor) and SB525334 (an ALK5 inhibitor) almost completely abolished the effects of *Pg*LPS, whereas KT5720 (a PKA inhibitor) had no effect (Fig. 1C). These results suggested that the induction of *Amtn* mRNA levels by LPS were mediated through the MEK1/2‐, PI3‐K‐ and ALK5‐involved Smad3 signaling pathways.

### *Pg*LPS increased mouse *Amtn* gene transcription

**Figure 2:**
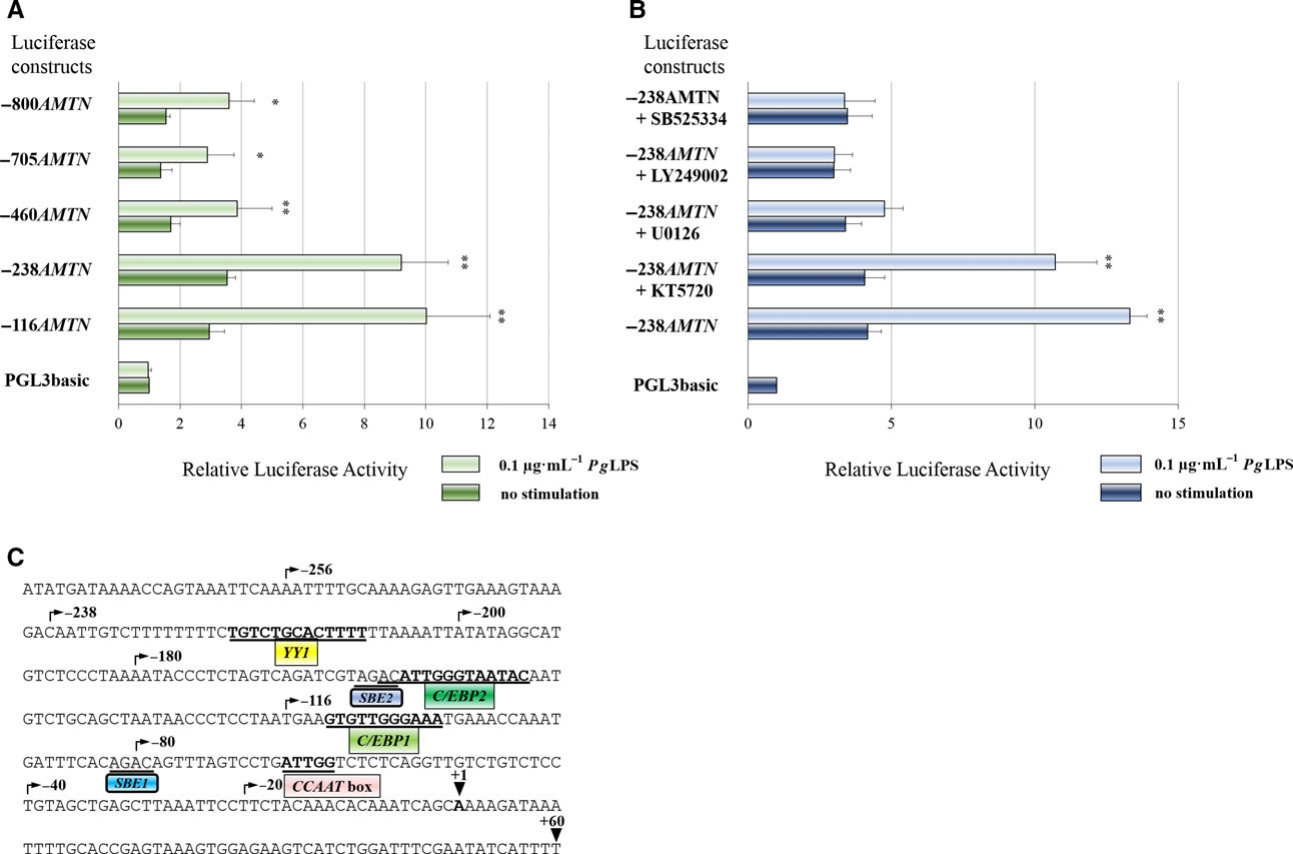
Regulation of *Amtn* promoter activity by *Pg* LPS in GE1 cells. (A) Transient transfections of GE1 cells in the presence or absence of *Pg* LPS (0.1 μg·mL ^−1^) for 24 h were used to determine transcriptional activities of chimeric constructs that included various regions of the *Amtn* gene promoter ligated to a luciferase reporter gene. Transcriptional activities obtained from three separate transfections with constructs PGL3‐basic, −116*AMTN* (−116 to +60), −238*AMTN* (−238 to +60), −460*AMTN* (−460 to +60), −705*AMTN* (−705 to +60) and −800*AMTN* (−800 to +60) have been combined, and the values are expressed with SE. *P <* *0.05 (*n *=* *3), **P <* *0.01 (*n *=* *3). (B) Effects of kinase inhibitors on transcriptional activation by *Pg* LPS. Transient transfection analysis of −238*AMTN* (−238 to +60) treated with *Pg* LPS (0.1 μg·mL ^−1^) for 24 h in GE1 cells is shown together with the effects of PKA (KT5720, 100 nm), MEK1/2 (U0126, 5 μm), PI3‐K (LY249002, 10 μm) and ALK5 (SB525334, 1 μm) inhibitors. The results obtained from three separate transfections were combined and the values expressed with SE. **P <* *0.01 (*n *=* *3). (C) Regulatory elements in the proximal mouse *Amtn* gene promoter. Sequences of mouse *Amtn* gene promoter (−290 to +60) and locations of response elements, such as inverted *CCAAT* box (−66 to −62), SBE1 (−82 to −79), *C/EBP1* (−113 to −103), *C/EBP2* (−157 to −144), which partially overlaps SBE2 (−159 to −156), and *YY1* (−221 to −209) are shown in the proximal promoter region of the mouse *Amtn* gene.

To determine how *Pg*LPS regulates *Amtn* gene transcription, transient transfection analyses were performed using chimeric constructs encompassing different regions of the mouse *Amtn* gene promoter ligated to a luciferase reporter gene. Transcriptional activities of the *Amtn* gene promoter constructs −116*AMTN* (−116 ~ +60), −238*AMTN* (−238 ~ +60), −460*AMTN* (−460 ~ +60), −705*AMTN* (−705 ~ +60) and −800*AMTN* (−800 ~ +60) were increased by *Pg*LPS (0.1 μg·mL^−1^) at 24 h (Fig. 2A). Basal activities of −116*AMTN* and −238*AMTN* constructs were 1.6‐ to 2.0‐fold higher compared with those of luciferase constructs including further upstream of the *Amtn* gene promoter. These results suggest that the *Amtn* promoter sequence between −238 and −460 may contain a suppressor element.

To determine the signaling pathways mediating the *Pg*LPS effects on *Amtn* gene transcription, kinase inhibitors were used as in the real‐time PCR analyses. *Pg*LPS‐induced −238*AMTN* promoter activity was inhibited by U0126, LY249002 and SB525334, but no effect was observed with KT5720, indicating an involvement of MEK1/2, PI3‐K/Akt and ALK5 in mediating the effects on *Amtn* gene transcription (Fig. 2B).

**Figure 3:**
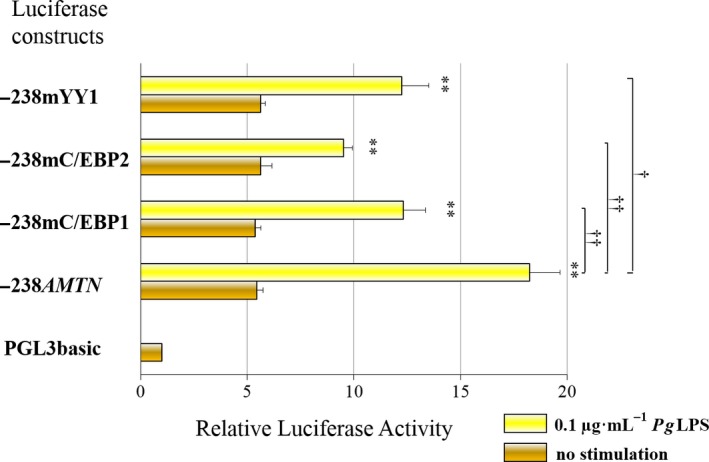
Regulation of *Amtn* gene promoter activities in mutation constructs of −238*AMTN* by *Pg* LPS in GE1 cells. Transient transfections of GE1 cells in the presence or absence of *Pg* LPS (0.1 μg·mL ^−1^) for 24 h were used to determine transcriptional activities of chimeric constructs that included mutant C/EBP1 (−238mC/EBP1; GTGTcGGtAAg), mutant C/EBP2 (−238mC/EBP2; ACATcGGaTAgTAC) and mutant YY1 (−238mYY1; TGcCTGCAgTcTT). Transcriptional activities of −238mC/EBP1, −238mC/EBP2 and −238mYY1 were lower than the basal level of −238*AMTN*, indicating that *C/EBP1*,* C/EBP2* and *YY1* response elements were affected by *Pg* treatment. The results obtained from three separate transfections were combined and the values expressed with SE. Comparison between no treatment and *Pg* LPS treatment: **P <* *0.01 (*n *=* *3); comparison of *Pg* LPS‐induced activity among each construct: ^†^ *P *<* *0.05 (*n *=* *3), ^††^ *P *<* *0.01 (*n *=* *3).

To identify *Pg*LPS response regions, we prepared −238*AMTN* mutant constructs that included 3 bp mutations in C/EBP1 (−238mC/EBP1), C/EBP2 (−238mC/EBP2) and YY1 (−238mYY1; Figs 2C and 3). Basal luciferase activities of −238mC/EBP1, −238mC/EBP2 and −238mYY1 constructs were slightly lower than the basal activity of −238*AMTN*. The transcriptional activities induced by *Pg*LPS were partially abrogated in the three mutation constructs, suggesting that C/EBP1, C/EBP2 and YY1 were response elements for *Pg*LPS (Fig. 3).

### Identification of response elements regulated by *Pg*LPS in GE1 cells

**Figure 4:**
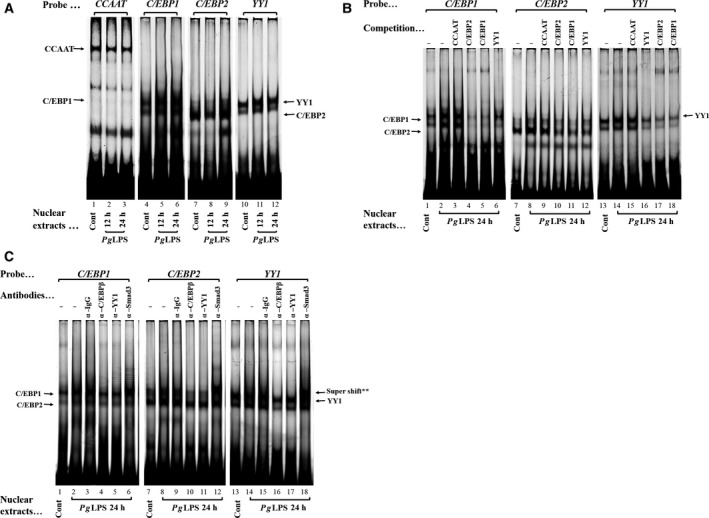
*Pg* LPS upregulates a nuclear protein that recognizes C/EBP1, C/EBP2 and YY1. (A) Cy5‐labeled double‐stranded *CCAAT* (−75 TTAGTCCTGATTGGTCTCTCAGG −53), *C/EBP1* (−120 CTAATGAAGTGTTGGGAAATGAAACC −95), *C/EBP2*2 (−163 TCGTAGACATTGGGTAATACAATGTC −138) and *YY1* oligonucleotides (−226 TTTTCTGTCTGCACTTTTTTAAAATT −200) were incubated for 20 min at RT with nuclear protein extracts (20 μg) obtained from GE1 cells treated without (lanes 1, 4, 7 and 10) or with *Pg* LPS at 0.1 μg·mL ^−1^ for 12 h (lanes 2, 5, 8 and 11) and 24 h (lanes 3, 6, 9 and 12). DNA–protein complexes were separated on 6% polyacrylamide gel in low‐ionic‐strength 0.5× TBE buffer and imaged by Typhoon Trio+ Variable Mode Imager. Black dotted lines in the figure indicate splice marks between separate gels. (B) Specific binding of nuclear proteins to *C/EBP1*,* C/EBP2* and *YY1*. Competition assays were performed using 40‐fold molar unlabeled oligonucleotides for *CCAAT* (lanes 3, 9 and 15), *C/EBP1* (lanes 5, 11 and 18), *C/EBP2* (lanes 4, 10 and 17) and *YY1* (lanes 6, 12 and 16). Cy5‐labeled double‐stranded *C/EBP1*,* C/EBP2* and *YY1* oligonucleotides were incubated for 20 min at RT with nuclear protein extracts (20 μg) obtained from GE1 cells treated without (lanes 1, 7 and 13) or with *Pg* LPS at 0.1 μg·mL ^−1^ for 24 h (lanes 2–6, 8–12 and 14–18). (C) Specific binding of nuclear proteins to *C/EBP1*,* C/EBP2* and *YY1*. Specific binding of nuclear proteins to the *C/EBP1*,* C/EBP2* and *YY1* elements. Cy5‐labeled double‐stranded *C/EBP1*,* C/EBP2* and *YY1* oligonucleotides were incubated with nuclear protein extracts (20 μg) for 20 min at RT obtained from GE1 cells treated without (lanes 1, 7 and 13) or with *Pg* LPS at 0.1 μg·mL ^−1^ for 24 h (lanes 2–6, 8–13 and 14–18). Supershift experiments were performed with 0.5 μg of antibodies against IgG (lanes 3, 9 and 15), C/EBPβ (lanes 4, 10 and 16), YY1 (lanes 5, 11 and 17) and Smad3 (lanes 6, 12 and 18). **Supershift of Smad3 added separately to each reaction. Anti‐YY1 antibody disrupted the formation of *YY1*–protein complexes (lane 17), and similarly, *C/EBP1*– and *C/EBP2*–protein complexes (lanes 5 and 11).

To determine whether *Pg*LPS altered nuclear proteins binding to the *C/EBP1*,* C/EBP2* and *YY1* elements, Cy5‐labeled double‐stranded oligonucleotides were incubated with equal amounts (20 μg) of nuclear proteins extracted from GE1 cells that were either not treated (Control) or treated with *Pg*LPS (0.1 μg·mL^−1^) for 12 and 24 h. After stimulation by *Pg*LPS, *C/EBP1*–protein complexes (Fig. 4A, lanes 5 and 6) and *YY1*–protein complexes (Fig. 4A, lanes 11 and 12) were increased at 12 and 24 h, and *C/EBP2*–protein complexes (Fig. 4A, lane 9) were increased at 24 h in GE1 cells. The nuclear protein binding to the inverted *CCAAT* sequence was not increased by *Pg*LPS treatment (Fig. 4A, lanes 1–3). To confirm the DNA–protein complexes represent specific interactions, competition gel shifts were carried out in which 40‐fold molar excess of *C/EBP1*,* C/EBP2* and *YY1* double‐stranded oligonucleotides reduced the amount of *C/EBP1*–, *C/EBP2*– and *YY1*–protein complex formation (Fig. 4B). Interestingly, non‐labeled 40‐fold molar excess of *C/EBP1*,* C/EBP2* and *YY1* oligonucleotides except for *CCAAT* competed with *C/EBP1*–, *C/EBP2*– and *YY1*–protein complexes. These results suggested that common transcription factors bind to the three elements and/or they interact with each other.

To further characterize the nuclear proteins in the complexes formed with the *C/EBP1*,* C/EBP2* and *YY1* elements, supershift assays were performed using specific antibodies. C/EBPβ is a member of the *CCAAT*/enhancer‐binding protein family of bZIP transcription factors 36. The addition of anti‐C/EBPβ and anti‐YY1 antibodies partially abrogated the *C/EBP1*–, *C/EBP2*– and *YY1*–protein complex formation (Fig. 4C, lanes 4, 5, 10, 11, 16, and 17). In order to confirm the possibility that Smad3 was involved in *C/EBP1–*,* C/EBP2*– and *YY1*–protein complexes, because the SBEs are located close to *C/EBP1*,* C/EBP2* and *YY1* elements (Fig. 2C), supershift assays were performed (Fig. 4C). Interestingly, anti‐Smad3 antibody induced visible supershifts on the *C/EBP1*–, *C/EBP2*– and *YY1*–protein complexes (Fig. 4C, lanes 6, 12, and 18).

**Figure 5:**
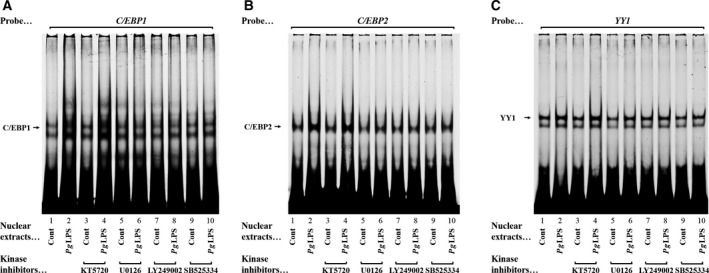
Effect of kinase inhibitors on (A) *C/EBP1*–, (B) *C/EBP2*– and (C) *YY1*–protein complex formation induced by *Pg* LPS (0.1 μg·mL ^−1^ for 24 h). Cy5‐labeled double‐stranded *C/EBP1*,* C/EBP2* and *YY1* oligonucleotides were incubated with nuclear protein extracts (20 μg) for 20 min at RT obtained from GE1 cells treated without (lanes 1, 3, 5, 7 and 9) or with *Pg* LPS (lanes 2, 4, 6, 8 and 10). The samples were treated with PKA (KT5720, 100 nm; lanes 3 and 4), MEK1/2 (U0126, 5 μm; lanes 5 and 6), PI3‐K (LY249002, 10 μm; lanes 7 and 8) and ALK5 (SB525334, 1 μm) inhibitors. DNA–protein complexes were separated on 6% polyacrylamide gel in low‐ionic‐strength Tris/borate buffer and exposed to an imaging plate for quantification using an imaging analyzer.

To support the results of transient transfection assays using several kinase inhibitors, we investigated the effects of kinase inhibitors on the DNA–protein complex formation with or without stimulation by *Pg*LPS. Increases in the *C/EBP1*–protein complex formation induced by *Pg*LPS (24 h) were abolished by U0126 (Fig. 5A, lanes 5 and 6), LY249002 (Fig. 5A, lanes 7 and 8) and SB525334 (Fig. 5A, lanes 9 and 10), but not by KT5720 (Fig. 5A, lanes 3 and 4). For increases in the C/EBP2– and YY1–protein complex formation, similar effects of U0126, LY249002 and SB525334 were observed (Fig. 5B,C, lanes 5–10). These results were consistent with the results of transient transfection assays using MEK1/2, PI3‐K and ALK5 inhibitors (Fig. 2).

**Figure 6:**
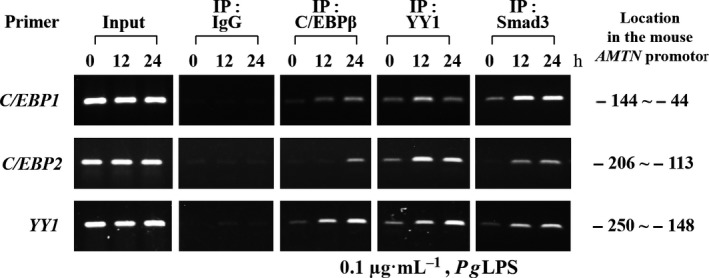
ChIP assay was performed using GE1 cells to investigate C/EBPβ, YY1 and Smad3 proteins bind to *C/EBP1*,* C/EBP2* and *YY1* elements in the mouse *Amtn* gene promoter. Binding of C/EBPβ to C/EBP1 and C/EBP2 was increased after 24 h stimulation by *Pg* LPS (0.1 μg·mL ^−1^), and C/EBPβ binding to YY1 was induced after 12 h stimulation by *Pg* LPS (0.1 μg·mL ^−1^). YY1 and Smad3 binding to C/EBP1, C/EBP2 and YY1 was induced after 12 h stimulation by *Pg* LPS (0.1 μg·mL ^−1^).

To further clarify the interaction between specific transcription factors, such as C/EBPβ, YY1 and Smad3, in the mouse *Amtn* gene promoter *in vivo*, chromatin immunoprecipitation assays (ChIP) assays were carried out. *Pg*LPS induced C/EBPβ binding to C/EBP1, C/EBP2 and YY1, and the YY1 binding to C/EBP1, C/EBP2 and YY1. Interestingly, Smad3 binding to C/EBP1, C/EBP2 and YY1 was increased by *Pg*LPS in a time‐dependent manner. The fragmented DNA–protein complexes were used for positive control (input) without using specific antibodies for immunoprecipitation (Fig. 6).

### Increase of protein levels of LPS‐induced transcription factors in GE1 cells

**Figure 7:**
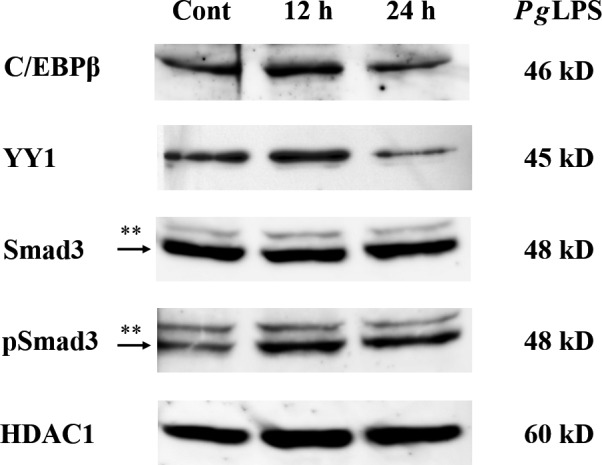
Change of C/EBPβ, YY1, Smad3 and pSmad3 nuclear proteins from GE1 gingival epithelial cells was examined with western blot analysis. After treatment with *Pg* LPS, protein levels of C/EBPβ and YY1 were increased at 12 h of treatment. Time‐dependent treatment with *Pg* LPS did not change Smad3 protein level; however, pSmad3 protein level was induced by treatment with *Pg* LPS at 12 and 24 h. Expression of HDAC1 was used as control. Densitometric analysis was performed (Table 3).

We next investigated effects of *Pg*LPS on the protein levels of transcription factors, such as C/EBPβ, YY1, Smad3 and pSmad3 which bind to C/EBP1, C/EBP2 and YY1 elements in the mouse *Amtn* gene promotor. *Pg*LPS increased C/EBPβ (1.29‐fold) and YY1 (1.40‐fold) protein levels at 12 h and pSmad3 protein levels at 12 (2.67‐fold) and 24 h (2.81‐fold), whereas Smad3 protein levels were not consistently changed by *Pg*LPS (Fig. 7 and Table 3).

**Figure 8:**
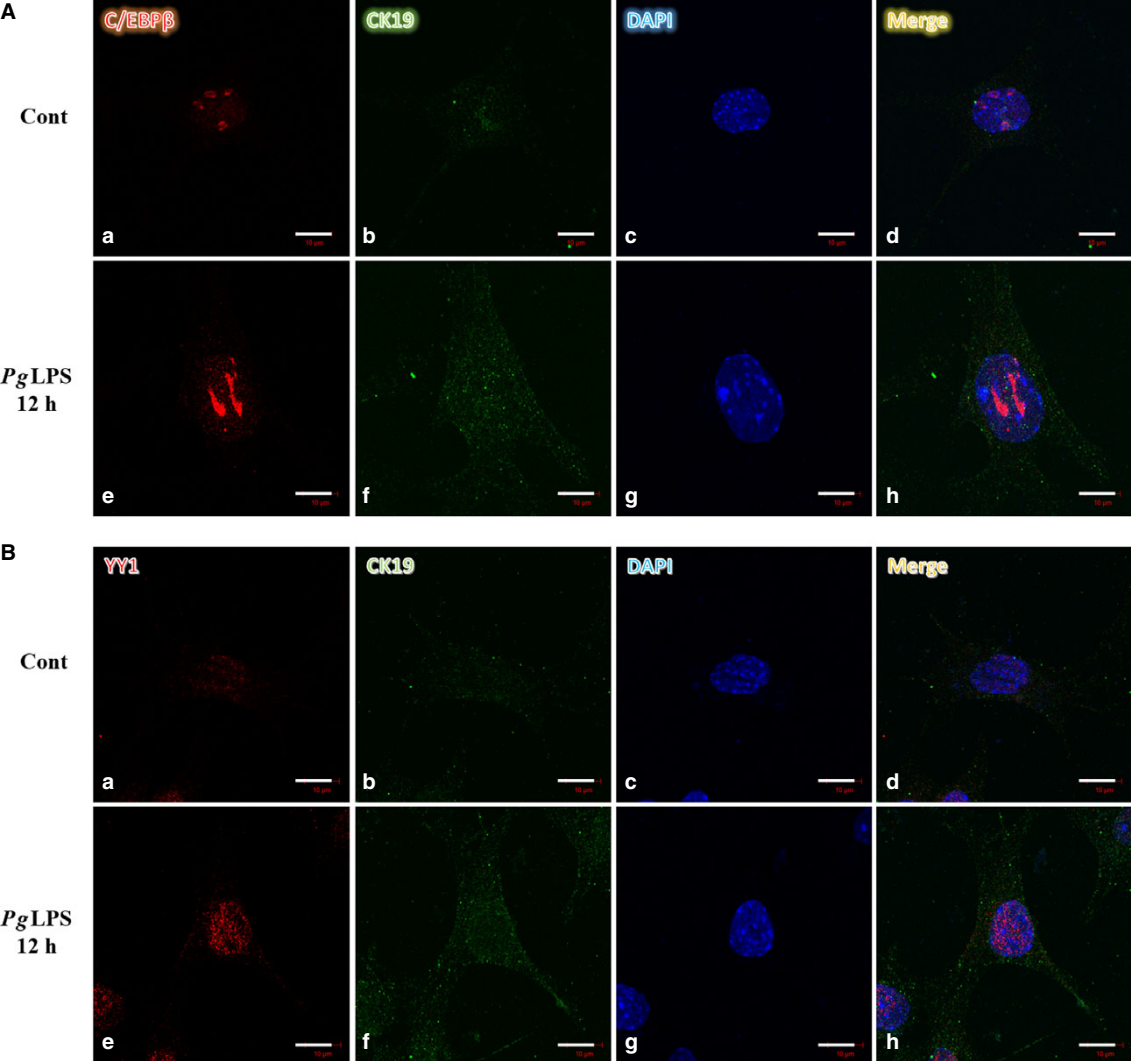
Expression of C/EBPβ (A) and YY1 (B) in GE1 gingival epithelial cells was examined with immunocytochemistry, after treatment with *Pg* LPS for 12 h (e–h), and no treatment (a–d; original magnification ×100.) Expression of C/EBPβ (Aa,e), YY1 (Ba,e), and CK19 (Ab,f, Bb,f). DAPI panel shows the location of the nucleus. Merged panel shows the results of combination of C/EBPβ, CK19 and DAPI panel or YY1, CK19 and DAPI panel. C/EBPβ and YY1 expression were induced by treatment with *Pg* LPS for 12 h (Ae,Be), consistent with the results of western blot analysis. CK19 expression was slightly increased by treatment with *Pg* LPS for 12 h (Af,Bf). Scale bars indicate 10 μm.

To elucidate the localization and protein expression of C/EBPβ and YY1, immunocytochemistry was carried out. C/EBPβ and YY1 protein levels in the nucleus were increased by treatment with *Pg*LPS for 12 h, consistent with the results of western blot (Fig. 8Ae,Be). Levels of CK19 protein, an inflammation marker, were slightly increased by *Pg*LPS (Fig. 8Af,Bf). These results suggest that *Pg*LPS‐induced C/EBPβ and YY1 and phosphorylation of Smad3 were associated with LPS‐induced *Amtn* gene transcription.

### C/EBPβ and YY1 directly interact with Smad3 in GE1 cells

**Figure 9:**
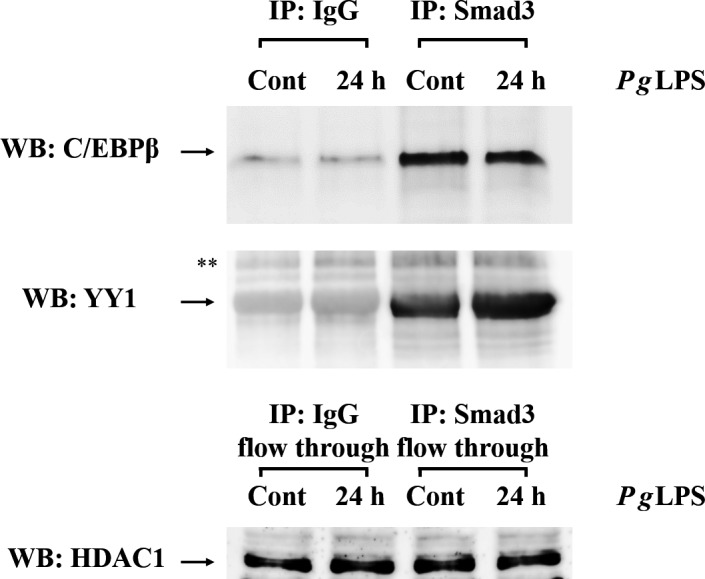
Interaction of C/EBPβ and Smad3 in the induction of *Amtn* gene expression by *Pg* LPS. Smad3‐precipitated C/EBPβ was detected, which indicated physical interaction. C/EBPβ and Smad3 complexes were not changed by *Pg* LPS treatment, compared with controls. Immunoprecipitation of IgG was performed as negative controls. Also shown is interaction of YY1 and Smad3 in the induction of *Amtn* gene expression by *Pg* LPS. Smad3‐precipitated YY1 was detected, which indicated physical interaction. YY1 and Smad3 complexes were remarkably induced by *Pg* LPS treatment. HDAC1 expression of flow through after immunoprecipitation did not change as controls. **Indicated non‐specific bands.

Smad3 is tightly associated with several transcription factors, and can promote or modulate their activities 36. Hence, we investigated whether the modulation of Smad3 activity by *Pg*LPS involves a stimulus‐dependent physical interaction. Immunoprecipitation analysis demonstrated that C/EBPβ in nuclear extract was co‐precipitated with Smad3 in GE1 cells; however, treatment with *Pg*LPS did not influence their interactions. In contrast, for the interaction between YY1 and Smad3, *Pg*LPS increased YY1 binding to Smad3 (Fig. 9). We performed western blot with HDAC1 antibody using flow‐through lysates after immunoprecipitation with anti‐IgG and anti‐Smad3 antibodies as the loading control; these results demonstrated that equal amounts of lysates were used for immunoprecipitation.

## Discussion

*Amtn* gene transcription is tightly regulated and is affected by the activity of inflammatory cytokines and their byproducts. *AMTN* can lead to pathologies including inflammation 5, 6, apoptosis 13, 14 and cancer 37. In this study we have shown that *Pg*LPS induced *Amtn* gene transcription that was mediated through C/EBPβ, YY1 and Smad3 binding to C/EBP1, C/EBP2 and YY1 elements, which was accompanied by physical interaction between C/EBPβ, YY1 and Smad3.

LPS could induce IL‐1β and TNF‐α production in THP‐1 cells 38, and IL‐1β and TNF‐α increased *Amtn* gene transcription in gingival epithelial cells 7, 8, 31. Similar to the results of these studies, we demonstrated that *Pg*LPS induced *Amtn* mRNA levels after 24 h treatment and upregulated mouse *Amtn* gene transcription via MEK1/2, PI3‐K and ALK5 signaling pathways in GE1 cells (Figs 1 and 2). It is known that LPS upregulates ALK5 expression in the induction of TNF‐α biosynthesis 39. Regulatory mechanisms of *Amtn* gene transcription by *Pg*LPS are consistent with those for IL‐1β and TNF‐α, indicating that IL‐1β and TNF‐α induced transcription factor binding to C/EBP1, C/EBP2 and YY1 elements 7, 8, 31 (Figs 2C, 4 and 6).

We confirmed that *C/EBP1*,* C/EBP2* and *YY1* elements are specific for LPS using mutant constructs of −238*AMTN*, in which basal transcriptional activity of −238*AMTN* was higher than the basal activity of −116*AMTN* (statistically significant difference, *P* < 0.05), whereas LPS‐induced −116*AMTN* and −238*AMTN* activities were almost the same, in spite of −238*AMTN* containing *YY1* and *C/EBP2* sites (Fig. 2A). The results of luciferase mutation assays using −238m*C/EBP1*, −238mC/EBP/2 and −238mYY1 suggest that these three response elements could be functional (Fig. 3). Further study is necessary to resolve the discrepancy.

Association with Smad2/3 signaling in aggressive periodontitis caused by *A. actinomycetemcomitans* infection was previously reported 40. Furthermore, we have shown that TGF‐β1‐induced *Amtn* gene transcription was mediated through Smad3 signaling pathways 13. Hence, we proposed that *Pg*LPS increased Smad3 binding to C/EBPβ and YY1, and thereby upregulated *Amtn* gene transcription. Smad3 is a critical transcription factor for the TGF‐β1 signaling pathway in tumor pathogenesis and contributes to cell growth, apoptosis, invasion and metastasis 41. Physical association of Smad3 and Smad4 with C/EBPβ and C/EBPδ provided repression of the function of EBPβ and C/EBPδ in inducing adipogenic differentiation of mesenchymal cells 36. In this study, the complexes of C/EBPβ and Smad3 were clearly detected by immunoprecipitation, whereas the amount of the complexes was not influenced by *Pg*LPS (Fig. 9A). Interestingly, physical interaction between YY1 and Smad3 was dramatically induced by *Pg*LPS (Fig. 9B). Considering to the results of C/EBPβ, YY1, Smad3 and pSmad3 protein levels after stimulation by *Pg*LPS (Fig. 7 and Table 3), LPS‐induced C/EBPβ, YY1 and pSmad3 directly bind to C/EBP1, *2* and YY1 elements, respectively, mediating their physical interaction with Smad3. In breast cancer cells, overexpression of C/EBPβ increased the protein expression levels of TGF‐β1 and pSmad3 and repressed Smad3 expression 42. Additionally, growth‐promoting activity of C/EBPβ has been observed in mammary epithelial cells 43, and Smad3 is required for the inhibition of adipogenesis by retinoic acid, mediated through the interference of C/EBPβ activity 44. These reports may represent an interaction of C/EBPβ with Smad3, or pSmad3 may be selectively and tightly regulated by cell types and numerous other biological processes. Thus, further research will address the mechanism and phosphorylation of Smad3 involved in the inflammation.

YY1 is a multifunctional zinc‐finger transcription factor belonging to the Polycomb protein family. This family is a group of homeobox gene receptors that regulate cell cycle and hematopoiesis, associated with cancer biology 31. YY1 is tightly associated with cancer; however, a relationship between *Amtn* and tumors has been reported in a few studies. *Amtn* expression led to positive staining of eosinophilic material in samples of adenomatoid odontogenic tumor 37, and detected in keratocystic odontogenic tumor cells 45. However, these studies did not mention a correlation between these tumors and YY1; therefore, further study is required to understand the relationship between YY1 and *Amtn* in initiation and metastasis of tumors.

In conclusion, this study showed that Complexes of *Pg*LPS‐induced C/EBP, YY1 and Smad3 are regulators of *Amtn* gene transcription via *C/EBP1*,* C/EBP2* and *YY1* elements in the mouse *Amtn* gene promoter. The C/EBPβ, YY1 and Smad3 complexes might be crucial player in the defense system of gingival epithelium against periodontal pathogens and inflammation.

## Conflicts of interest

The authors declare no conflict of interest.

## Author contributions

We declared that all the listed authors have participated actively in the study and all meet the requirements of the authorship. YN and YO designed the study and wrote the protocol. RK, YI, KN, MY, TK and BG performed research/study. YN and YO managed the literature searches and analyses. YN undertook the statistical analysis. YN and YO wrote the first draft of the manuscript.
